# Expanding the transgene expression toolbox of the malaria vector *Anopheles stephensi*

**DOI:** 10.1111/imb.12953

**Published:** 2024-08-11

**Authors:** Joshua Southworth, Estela Gonzalez, Katherine Nevard, Mireia Larrosa‐Godall, Luke Alphey, Michelle A. E. Anderson

**Affiliations:** ^1^ Department of Biosciences Durham University Durham UK; ^2^ Arthropod Genetics The Pirbright Institute Pirbright UK; ^3^ Department of Biology University of York York UK; ^4^ York Biomedical Research Institute University of York York UK; ^5^ Present address: Animal and Plant Health Agency Addlestone UK

**Keywords:** *Anopheles stephensi*, genetic biocontrol, malaria, *piggyBac*, transgene expression

## Abstract

*Anopheles stephensi* Liston, 1901 (Diptera: culicidae) is a competent vector of *Plasmodium falciparum* (Haemosporida: plasmodiidae) malaria, and its expansion in the African continent is of concern due to its viability in urban settings and resistance to insecticides. To enhance its genetic tractability, we determined the utility of a ~2 kb *An. stephensi lipophorin* (*lp*) promoter fragment in driving transgene expression. Lipophorin genes are involved in lipid transport in insects, and an orthologous promoter in *An. gambiae* (AGAP001826) was previously demonstrated to successfully express a transgene. In the present study, we qualitatively characterised the expression of a ZsYellow fluorescent marker protein, expressed by *An. stephensi lp* promoter fragment. Our study indicated that the *lp* promoter fragment was effective, generating a distinct expression pattern in comparison to the commonly utilised 3xP3 promoter. The *lp*:ZsYellow fluorescence was largely visible in early instar larvae and appeared more intense in later instar larvae, pupae and adults, becoming especially conspicuous in adult females after a blood meal. Different isolines showed some variation in expression pattern and intensity. Aside from general transgene expression, as the *lp* promoter produces a suitable fluorescent protein marker expression pattern, it may facilitate genotypic screening and aid the development of more complex genetic biocontrol systems, such as multi‐component gene drives. This study represents an expansion of the *An. stephensi* genetic toolbox, an important endeavour to increase the speed of *An. stephensi* research and reach public health milestones in combating malaria.

## Graphical abstract

- *An. stephensi* lipophorin (*lp*) gene fragment drives expression of the ZsYellow marker protein in the fat body of aquatic and adult life stages of *An. stephensi* validating its utility for expression in these tissues.
- The *lp*‐driven expression is distinguishable from the commonly utilised 3xP3 promoter, suggesting that both promoters could be used effectively in tandem for marker expression in more complex genetic systems.
- The successful implementation of the *An. stephensi lp* promoter in driving transgene expression highlights its potential for expanding the *An. stephensi* genetic toolbox through its use in genetic research and in the development of genetic biocontrol systems.

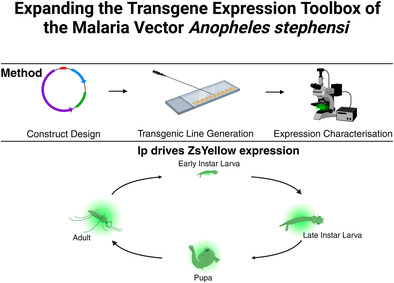

## INTRODUCTION

Anopheline mosquitoes are vectors of malaria, a disease caused by various *Plasmodium* parasite species that can affect a wide range of animals, including humans. Despite extensive efforts to reduce the global disease burden attributed to human malaria, it continues to cause over 500,000 recorded deaths annually, spanning more than 85 countries (World Health Organization, 2022). The vast majority of these deaths occur in children aged under 5 years. Hindering efforts to eradicate malaria is the emerging resistance of *Plasmodium* parasites to some treatments and the resistance of its vectors to insecticides (N'Guessan et al., 2007; Yared et al., 2020; Zhu et al., 2022). Additionally, the invasion of mosquito species into new geographical regions is projected to challenge vector control campaigns in the near future. Specifically, *Anopheles stephensi* Liston 1901, native to parts of Asia, has invaded Africa. The first instance of its presence was recorded in Djibouti in 2012 (Faulde et al., 2014), since then it has spread to many countries on the African continent (Ahmed et al., 2021; Carter et al., 2018; Sinka et al., 2020). This mosquito species is a competent vector of *Plasmodium falciparum* malaria (Hemming‐Schroeder & Ahmed, 2022) and is of particular concern due to its viability in urban environments (Lehmann et al., 2023; Vipin et al., 2010). Additionally, resistance against multiple common insecticides, including carbamates and pyrethroids, has been recorded in this species (Yared et al., 2020).

A proactive, integrated approach is clearly the most effective strategy to achieve further malaria eradication milestones. Beyond supporting the development of other control methods, understanding the genetics of *An. stephensi* holds promise for controlling this malaria vector through the development of genetic biocontrol systems. Therefore, characterising genetic toolbox components of non‐model organisms like *An. stephensi* is crucial to increase their genetic tractability and expedite malaria research efforts.

In this study, we determined the suitability of the *An. stephensi lipophorin* (*lp*) promoter region as a regulator of transgene expression. The lipophorin gene encodes a precursor to apolipophorin‐I and apolipophorin‐II, proteins that constitute lipophorin complexes. Lipophorins have various roles, primarily based upon their capacity to transport lipids throughout the bodies of insects (Canavoso et al., 2003). In *An. gambiae*, reverse transcriptase polymerase chain reaction (RT‐PCR) analysis confirmed the presence of *lp* transcripts at all key developmental stages. Furthermore, *lp* gene expression exhibited tissue specificity, with transcripts detected in the fat body but absent in the ovaries and Malpighian tubules of sugar‐fed females (Marinotti et al., 2006). Previously, a 1.6 kb *lipophorin* (AGAP001826) promoter fragment was used to drive tdTomato reporter gene expression in *An. gambiae*, facilitating transgene expression in the fat body throughout most of the mosquito life cycle. Additionally, expression was observed in the guts of neonate larvae that had yet to develop fat bodies (Volohonsky et al., 2015). The previously observed transgene expression pattern, driven by the *An. gambiae lp* promoter fragment, made the *An. stephensi* ortholog a suitable candidate for characterisation. Here, we qualitatively assessed the expression of a ZsYellow (ZsY) fluorescent protein when driven by a 2 kb *lp* promoter fragment in *An. stephensi*.

## RESULTS

### *The* lp *promoter sequence is active in early life stages of* An. stephensi

**FIGURE 1:**
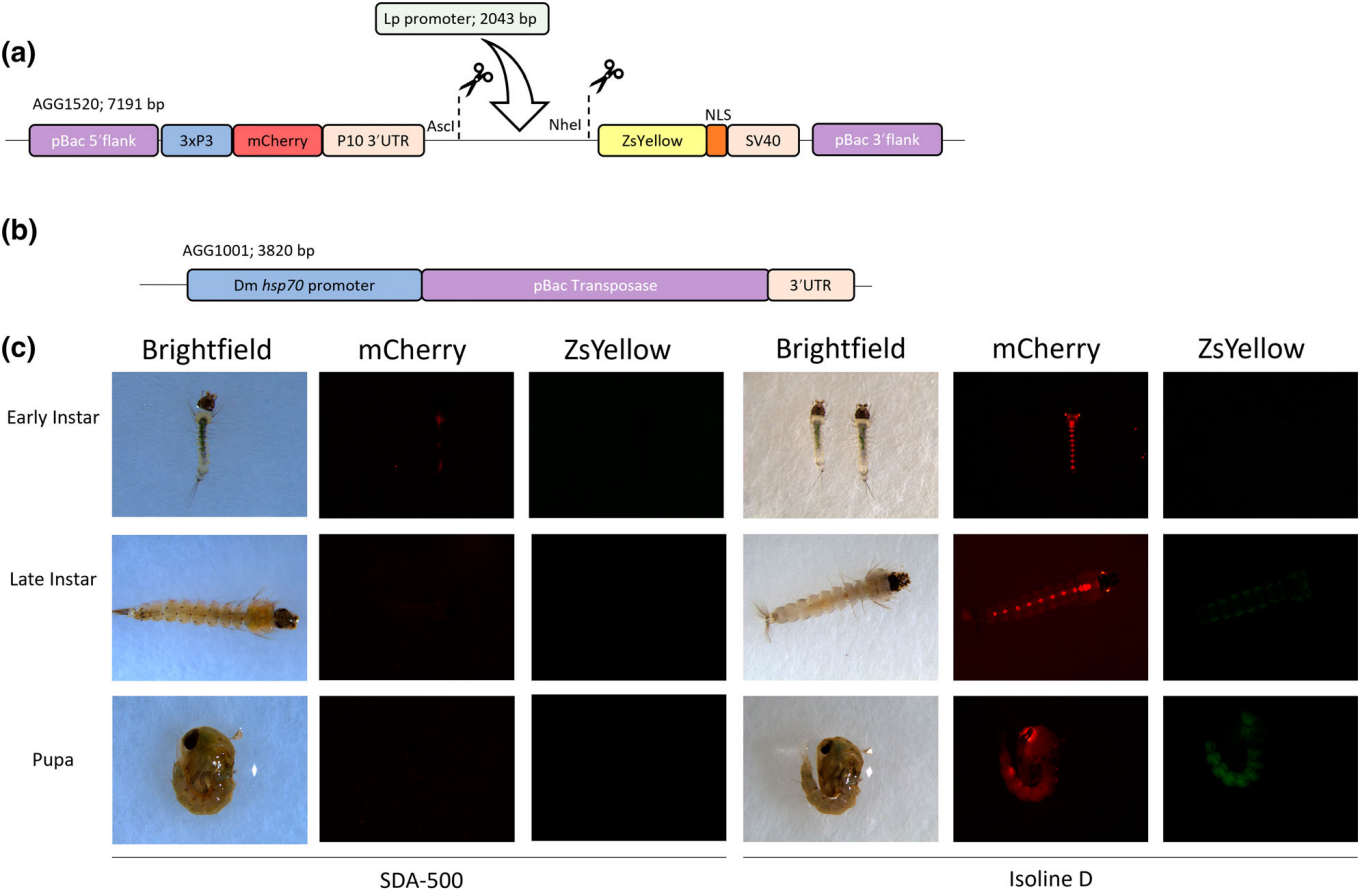
*Anopheles stephensi* AGG2282 individuals exhibit 3x3P:mCherry and *lp*:ZsYellow fluorescence across various aquatic life stages. Schematic of the plasmids used; AGG1520 plasmid carrying the 3xP3‐mCherry cassette and the ZsYellow marker in which the *An. stephensi* 2 kb *lipophorin* promoter fragment was inserted for expression of the latter. Dashed lines designate the restriction enzymes used for cloning (a). AGG1001 *piggyBac* transposase helper plasmid (b). Individuals from isoline D and wild‐type (SDA‐500) imaged in white light, mCherry filter and ZsY filter. Camera settings for individual images detailed in Table S2. Dm hsp70, *Drosophila melanogaster heat‐shock protein 70*; NLS, nuclear localization signal; P10 3'UTR, *Autographa californica nucleopolyhedrovirus* (AcMNPV) P10 3′UTR; pBac, *piggyBac*; SV40, *Simian virus 40* 3'untranslated region.

A total of 399 G_0_ embryos were injected, of which 24 survived to adulthood for crossing. The 12 surviving G_0_ males were crossed to 60 SDA‐500 females as a pool, and the 12 G_0_ females were crossed to 22 SDA‐500 males in a second pool. G_1_ individuals were screened to identify those that exhibited mCherry fluorescence, indicating the *piggyBac*‐mediated insertion of the construct, encoding both 3xP3:mCherry and *lp*:ZsYellow. A total of 101 transgenic G_1_ larvae were identified, with 92 displaying a similar expression pattern (90%). Ten G1 individuals were selected for single pair crossing and generation of isolines. Of these, six (A–F) displayed this common phenotype, the other four (G–J) were selected as displaying some deviation from the typical phenotype. This is likely due to different insertion sites of the construct, possibly also multiple insertions within the G_1_ parent, both being phenomena commonly observed with *piggyBac*‐mediated transgenesis. A total of 373 G_1_ individuals were screened from pool A, of which 101 were positive for both mCherry and ZsYellow fluorescence. G_1_ screening was discontinued thereafter due to the large quantity of positive individuals (Table S3). Although the isolines were derived from different G_1_ individuals, they do derive from the same pool of G_0_ parents which does not preclude the possibility of some isolines sharing the same insertion site. The few variations observed differed in their degrees of extremity, primarily consisted of marked differences in fluorescence intensity, but in some cases in the localisation of the *lp*‐ or 3xP3‐expressed fluorescence. Individuals from four isolines (A, D, G, I) were selected for futher study across all life stages with A and D representing the most commonly observed expression pattern and G and I representing variations in phenotype. The mCherry expression patterns were congruent with those of previous characterisations of 3xP3‐driven transgene expression in *An. stephensi*. This entailed fluorescence from the notochord and developing eyes of early and late‐instar larvae, with pupae exhibiting intense fluorescence around their eyes (Figure 1c Isoline D, mCherry). Additionally, these individuals displayed ZsYellow fluorescence in a distinct pattern, validating the *An. stephensi lp* promoter sequence as a capable driver of transgene expression (Figure 1c, isoline D, ZsY).

**FIGURE 2:**
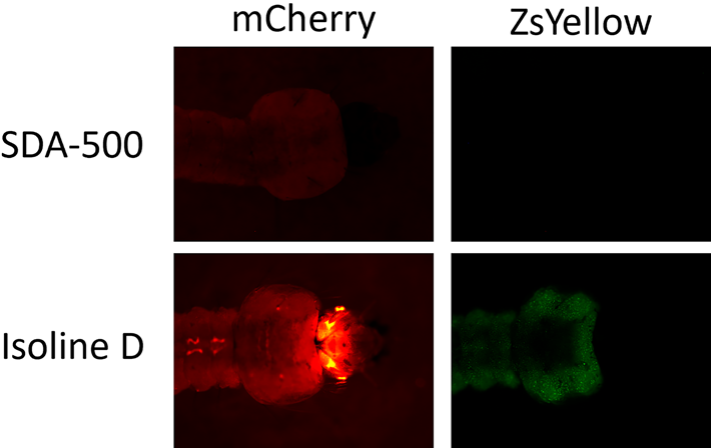
Fluorescence in late‐instar transgenic *Anopheles stephensi* larvae indicates distinct expression patterns in the head. mCherry and ZsYellow fluorescence in wild‐type (SDA‐500) and *lp*:ZsY isoline D. Camera settings for individual images detailed in Table S2.

Fluorescence from *lp*:ZsY was dim yet apparent under high magnification across the bodies of early instar individuals. Late‐instar larvae exhibited *lp*:ZsY fluorescence throughout their fat bodies (Figures 1c and 2; Figure S1), the intensity of which diminished at their anterior and posterior ends. Pupae exhibited *lp*:ZsY fluorescence throughout most of the body, including the pupal head, although no fluorescence was apparent in or directly around the eyes (Figure 1c, isoline D).

Viewing late‐instar larvae at a high magnification, the punctate pattern of *lp*:ZsY fluorescence is apparent, contrasting with the more uniform presentation of 3xP3:mCherry fluorescence (Figure 2). The punctate pattern likely arises from the nuclear localization signal (NLS) encoded directly downstream of the ZsY protein. Notably, fluorescence from 3xP3:mCherry is distinctly intense in the larval eye and absent in the case of *lp*:ZsY (Figure 2). In general, both *lp*‐ and 3xP3‐driven fluorescence was more apparent when observing larvae at higher magnifications.

### lp:ZsY expression increases after blood feeding

**FIGURE 3:**
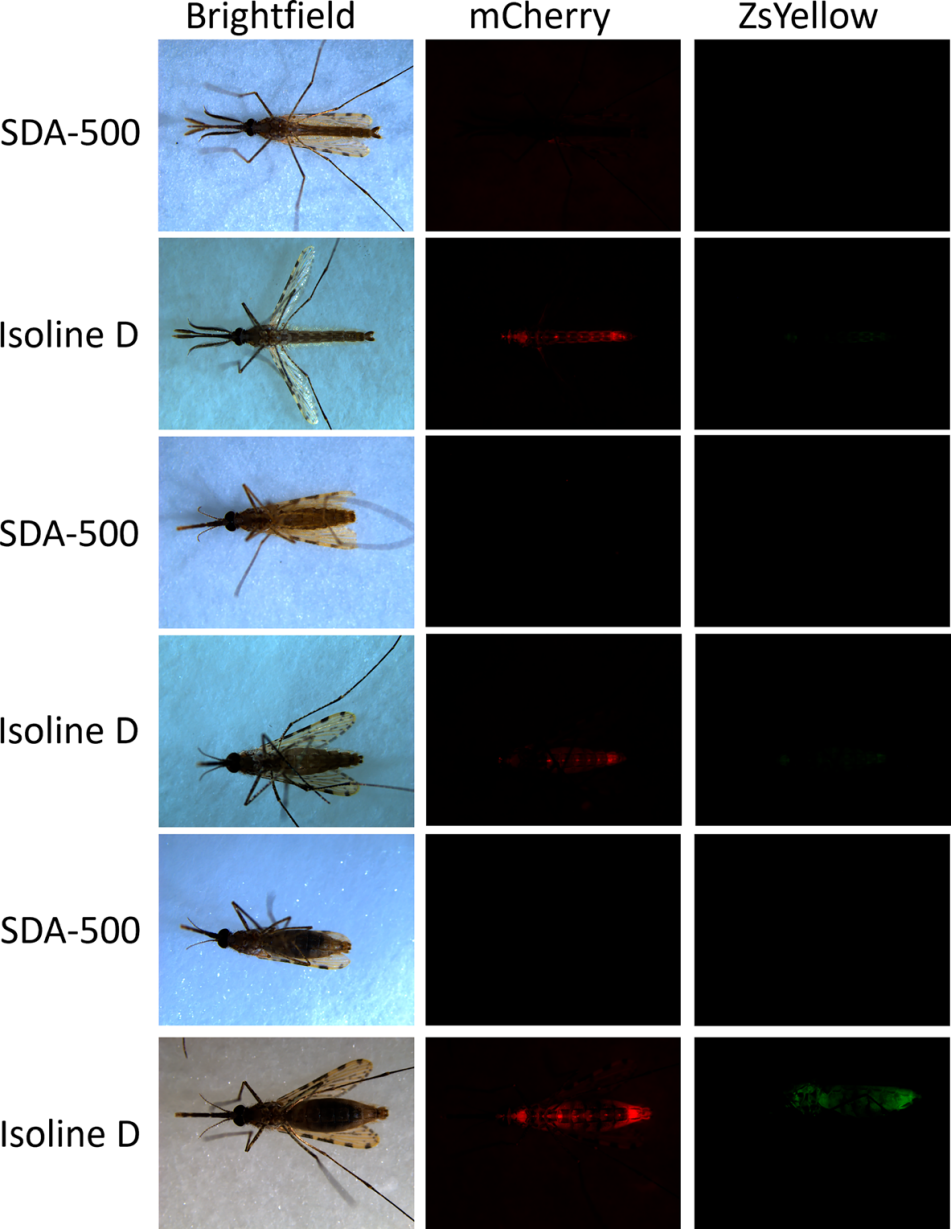
Transgenic *Anopheles stephensi* adults, express *lp*:ZsY and 3x3P:mCherry. SDA‐500 or Isoline D males (top 2 rows), females (middle 2 rows) and 24 h post‐blood meal (PBM) females (bottom 2 rows) under white light, mCherry or ZsYellow filters. Camera settings are detailed in Table S2.

In both male and female adults, 48 h post‐eclosion, *lp*:ZsY fluorescence was most apparent in the thorax and the terminal segments of the abdomen when viewed ventrally (Figure 3). In females, an increase in *lp*:ZsY fluorescence intensity was evident throughout the body 24 h post‐blood meal (PBM). The intensity of 3xP3:mCherry expression also seemed to increase 24 h PBM, and this was observed in several isolines (Figure S2). This could be a result of blood meal induced upregulation of expression of the *lp* promoter trans‐activating the 3xP3 promoter and/or aberrant expression of the 3xP3. Alternatively, this apparent increase in intensity could be caused by compression of fluorescent tissue by the distention of the midgut.

## EXPERIMENTAL PROCEDURES

### An. stephensi *maintenance*

*Anopheles stephensi* from the SDA‐500 strain (wild‐type [WT]) mosquitoes, kindly provided by Andrew Blagborough, were maintained in an environmental chamber at 28°C, 85% relative humidity (RH) and under a 14/10 day/night cycle. Larvae were reared in reverse osmosis (RO) water. Neonate larvae were fed with resuspended dry food (Seramicron, Sera), and later instars were fed fish food pellets (ExtraSelect, Su‐Bridge Pet Supplies Ltd). Adults were fed 10% sucrose solution *ad libitum*. Defibrinated horse blood (TCS Biosciences) was provided as a blood meal using a Hemotek (Hemotek, Ltd) device. All mosquitoes were maintained in an insectary, and the work was approved by Biological Agents and Genetic Modification Safety Committee (BAGMSC) at The Pirbright Institute.

### Construct design and synthesis

The *An. gambiae lp* gene sequence (AGAP001826) was used to identify the putative *An. stephensi* ortholog (ASTE006592). Primers LA7703 and LA7704 (Table S1) were designed to amplify ~2000 bp upstream of the *lp* start codon. This fragment was amplified using Q5 polymerase (New England Biolabs, NEB) from genomic DNA isolated from *An. stephensi* SDA‐500 strain using the Machery‐Nagel NucleoSpin Tissue kit following the manufacturer's instructions.

The *lp* promoter fragment was cloned into an existing plasmid (AGG1520, Figure 1a) containing piggyBac flanks, mCherry fluorescent marker under the expression of the 3xP3 promoter and the ZsYellow open reading frame (ORF). The *lp* promoter fragment was digested with *Asc*‐I and *Nhe*I‐HF and ligated (T4 DNA ligase, NEB) into the same sites of the AGG1520 plasmid, upstream of the ZsYellow ORF. The ligation mixture was transformed into SURE2 cells (Agilent). Colony PCR was performed using primers LA6798 and LA7721 (Table S1) and DreamTaq polymerase (Fisher Scientific) to identify successful colonies. Positive colonies were miniprepped using the Plasmid Mini kit (Machery‐Nagel) and confirmed by Sanger sequencing. The final plasmid (AGG2282, Figure 1a) Genbank accession number OR829553.1.

### Transgenic line establishment

Microinjections on WT embryos were performed as previously described (Catteruccia et al., 2005). The microinjection mix was composed of 300 ng/μL AGG1001 hsp70‐piggyBac (Handler & Harrell, 1999) as helper (Figure 1b), 300 ng/μL AGG2282 and 1× injection buffer (Coates et al., 1998), made up with endotoxin free H_2_0.

The G_0_ injection survivors were reared as described in the *An. stephensi* maintenance section above. Males and females that reached adulthood were crossed to 3–5 day old virgin WT individuals of the opposite sex at ratios of 1:5 and 1:2, respectively. In total, 12 G_0_ females were pooled into one cross and 12 G_0_ males into another, producing pools A and B, respectively. Mosquitoes were allowed to mate for at least 2 days, after which the females were offered blood meals. The G_1_ eggs were collected, hatched and reared for screening as L3–L4 instar larvae under a Leica MZ165C fluorescence microscope, using appropriate mCherry and ZsYellow filters From the surviving G_1_ adults, 10 of the 101 individuals positive for both mCherry and ZsYellow fluorescence were individually crossed to SDA‐500 to generate isolines designated A–J (Table S3).

### *Fluorescence imaging of transgenic* An. stephensi *life stages*

Images were taken using a Leica MZ165C fluorescence microscope with LEICA DFC7000T‐11,547,106 camera attached using the LAS software. Individuals were imaged through mCherry or ZsYellow fluorescence filters, or white light, as indicated. Further details of camera settings can be found in Table S2.

## DISCUSSION

In this study, we demonstrated the effectiveness of *An. stephensi lp* promoter fragment in driving transgene expression. When utilised to promote the expression of a fluorescent protein, ZsYellow, fluorescence was visible across all aquatic *An. stephensi* life stages. Additionally, *lp*‐driven ZsYellow expression was observable in both male and female adults, though the intensity of fluorescence in unfed females appeared to be particularly weak and was limited to the terminal abdominal segments.

Utilising a larger genomic fragment for the promoter could potentially enhance the relatively dim expression observed here. It should also be noted that in our experience the ZsYellow fluorescent protein can appear relatively dimmer than other fluorescent proteins commonly used as transgene markers. The genomic loci of the inserted constructs were not molecularly determined in this study and it is possible that some isolines may contain multiple insertions of the transgene. For applications where a single insertion is required further verification would be necessary, however, the expression pattern of this promoter fragment has useful characteristics. Expressed alongside mCherry, expressed by the previously characterised 3xP3 promoter, the expression patterns produced by each promoter were distinct and could be distinguished in the same individual. Exemplifying this, *lp*:ZsY expression was absent from the eyes of larvae and pupae, whereas 3xP3:mCherry produced intense expression in the eyes of individuals in their aquatic life stages. Furthermore, *lp*‐driven expression presented as a punctate pattern across the fat body of individuals, whereas 3xP3‐driven fluorescence appeared in a more solid pattern, primarily in the notochord of individuals. Although the observed punctate pattern of ZsY is likely due to the presence of the NLS, and not a specific property of *lp*‐driven expression, these findings indicate the merit of the *An. stephensi lp* promoter sequence for driving marker protein expression for genotypic screening purposes in this mosquito species. The dim fluorescence observed during early instars should not hinder conventional genotypic screening, typically performed with later instar larvae that are more robust against physical manipulations. However, the dim fluorescence may be an indication that this promoter fragment is not suitable for applications where expression is required in early larvae, conversely it may be desirable where expression of a transgene product might be deleterious to early larvae.

A previous study by Volohonsky et al. (2015) characterised a 1.6 kb fragment of an *An. gambiae lp* promoter sequence for the expression of a tdTomato reporter protein. Similar to our observations, tdTomato expression was visible in the fat body of fourth instar larvae and adults (a characterisation of pupae was not reported in this study). Where the resolution of our study was limited to defining the larval mosquito stages as ‘early,’ corresponding to neonate, L1 and L2 larvae, and ‘late,’ corresponding to L3 and L4 larvae, Volohonsky et al. (2015) specifically characterised *lp*‐driven expression in neonate larvae. They found that expression was absent in the neonate larva fat body, though some fluorescence was present in the digestive tract. Mosquito larvae develop their fat body over time (Volohonsky et al., 2015), which explains the lack of *lp*‐driven expression observable in neonate *An. gambiae* larvae and the relatively weak intensity of fluorescence in the early instar of *An. stephensi* larvae screened in our study. This low level of expression in early larvae could limit the utility of this promoter in studies that use automated genotypic screening, for example, using large particle flow cytometry. However, this remains to be elucidated.

The distinct expression patterns produced by *lp* and 3xP3 suggest their feasibility for joint use. In cases where more overlap is apparent between *lp*‐ and 3xP3‐regulated expression, the conspicuous fluorescence observed in the larval eye, and its marked absence from the entire larval head under *lp* regulation, enables facile detection of 3xP3‐related fluorescence in individuals encoding both promoters for marker expression (Figure 2). It is important to note that different fluorescent marker proteins naturally have different stabilities and intensities of fluorescence, so this study cannot be used as a resource to compare the levels of transgene expression achievable between the *An. stephensi lp* and 3xP3 promoters.

Expanding on the variety of promoter elements available, and used in conjunction with marker proteins, means more genetic elements can be tracked in an individual without the requirement of molecular genotyping. In the case of anopheline mosquitoes, this can aid general explorative research efforts but also the development of more complex genetic biocontrol systems. For example, self‐limiting CRISPR/Cas9‐based homing endonuclease gene drives can consist of multiple genetic elements, segregated across the mosquito genome (Akbari et al., 2015). Additionally, hypothetical daisy chain gene drives of this kind utilise an indefinite quantity of genetic elements to fine tune their spatiotemporal action (Noble et al., 2019). These would ideally be tracked by fluorescent markers during their development, a clear case where the availability of multiple promoters that facilitate distinct expression would be desirable.

Considering applications beyond the expression of marker proteins for genotypic screening, the *An. stephensi lp* promoter sequence is a promising tool for contexts where transgene expression in the mosquito fat body is desired. Exemplifying this, a recent study expressed a single‐chain anti‐*P. falciparum* antibody, under the regulation of the native *lp* promoter in *An. coluzzii* as part of a population modification gene drive (Green et al., 2023). This decreased the ability of the transgenic mosquitoes to transmit *P. berghei*, expressing a *P. falciparum* sporozoite protein, to mice. This highlights the utility of the *lp* promoter in expressing anti‐pathogen effectors in mosquitoes, conducive to vector control efforts. Additionally, the localisation of *lp*‐driven expression in the fat body gives it utility in use cases where the expression of proteins intended to be secreted into the haemolymph is required. The potential blood meal inducible expression in adult females could also be an attractive phenotype for many applications, although this would require further optimisation of insertion site and potentially use of a larger promoter fragment to ensure this phenotype was replicated.

Overall, the presented characterisation of the *An. stephensi lp* promoter has successfully added to the toolbox of genetic elements that can be utilised for *An. stephensi* research. Although a wealth of tools is available for analysing and modifying model organisms, the same is not true for others. Despite this, the study of many non‐model organisms can be beneficial to public health outcomes. Mosquito species that spread diseases are a key example where this is the case. Even if research done in comparable model organisms, like *Drosophila*, can be transferable, differences in the biology of these organisms mean this is not always the case, especially in the case of applied research for vector control. Expanding the genetic toolbox of a species is a clear way to increase the speed and ease with which research can be performed. In the case of anopheline mosquitoes, this is highly desirable for achieving public health milestones associated with their transmitted diseases.

## AUTHOR CONTRIBUTIONS

**Joshua Southworth:** Writing – original draft; investigation; writing – review and editing; visualization. **Estela Gonzalez:** Supervision; writing – original draft; writing – review and editing; investigation; methodology. **Katherine Nevard:** Writing – review and editing; investigation. **Mireia Larrosa‐Godall:** Writing – review and editing; investigation. **Luke Alphey:** Methodology; funding acquisition; supervision; writing – review and editing. **Michelle A. E. Anderson:** Conceptualization; writing – review and editing; supervision; methodology.

## FUNDING INFORMATION

This publication is based on research funded in part by the Bill & Melinda Gates Foundation. The findings and conclusions contained within are those of the authors and do not necessarily reflect positions or policies of the Bill & Melinda Gates Foundation. This work was also supported by strategic funding from the UK Biotechnology and Biological Sciences Research Council (BBSRC) to The Pirbright Institute (BBS/E/I/00007033, BBS/E/I/00007038 and BBS/E/I/00007039).

## CONFLICT OF INTEREST STATEMENT

Luke Alphey is an adviser to Synvect Inc and Biocentis Ltd, with equity and/or financial interest in those companies. The other authors declare that they have no competing interests.

## ETHICS STATEMENT

All experiments performed for this study were reviewed and approved by the Biological Agents and Genetic Modification Safety Committee (BAGMSC) at The Pirbright Institute.

## Supporting information


**Figure S1.** *An. stephensi* larvae from various isolines of the *lp*:ZsY transgenic line. SDA‐500 and transgenic larvae under white light, mCherry or ZsY filter. Camera settings are detailed in Table S2.
**Figure S2.** Transgenic *An. stephensi* adults, express *lp*:ZsY and 3x3P:mCherry. Wild‐type (SDA‐500) and individuals from isolines A, D, G and I under white light, mCherry and ZsY filters before and 24 h post‐blood meal (PBM). Camera settings are detailed in Table S2.
**Table S1.** List of primers used in the study.
**Table S2.** Visual magnification and exposure (ms) settings for image acquisition.
**Table S3.** Embryonic microinjections.

## Data Availability

All data generated for this study is included in the manuscript and supplementary files and sequences can be found in the NCBI database.
