# Supplementary material for: Expanding the transgene expression toolbox of the malaria vector Anopheles stephensi
Source: Insect Mol Biol. 2024 Aug 11;34(1):104–10. doi: 10.1111/imb.12953 (PMC11705503; doi:10.1111/imb.12953)
Supplement: Supplementary file 1 — Figure S1. An. stephensi larvae from various isolines of the lp:ZsY transgenic line. SDA‐500 and transgenic larvae under white light, mCherry or ZsY filter. Camera settings are detailed in Table S2. Figure S2. Transgenic An. stephensi adults, express lp:ZsY and 3x3P:mCherry. Wild‐type (SDA‐500) and individuals from isolines A, D, G and I under white light, mCherry and ZsY filters before and 24 h post‐blood meal (PBM). Camera settings are detailed in Table S2. Table S1. List of primers used in the study. Table S2. Visual magnification and exposure (ms) settings for image acquisition. Table S3. Embryonic microinjections. [file IMB-34-104-s001.docx]

**Supplemental Material**

**
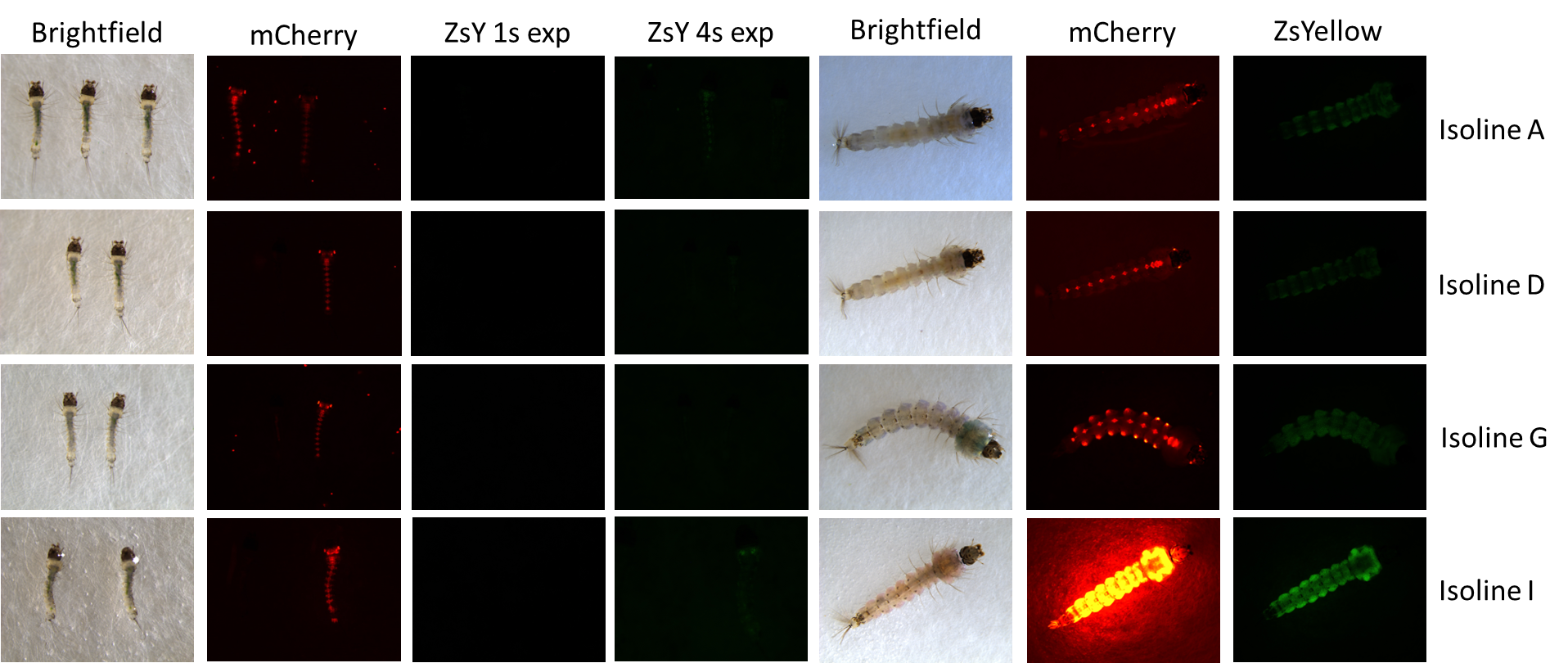
**

**Figure S1. An. stephensi larvae from various isolines of the lp:ZsY transgenic line.** SDA-500 and transgenic larvae under white light, mCherry or ZsY filter. Camera settings are detailed in Table S2.


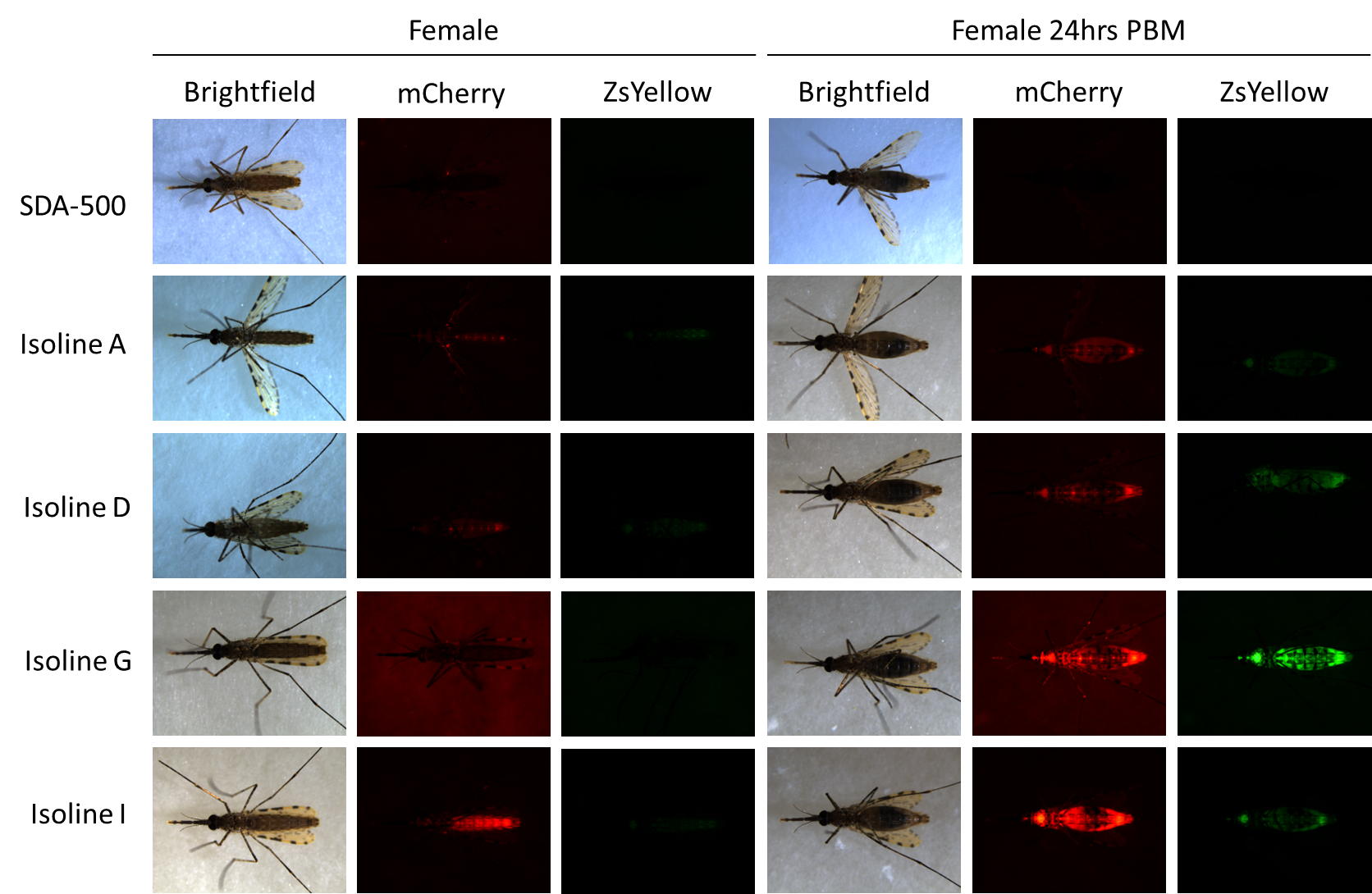


**Figure S2. Transgenic An. stephensi adults, express lp:ZsY and 3x3P:mCherry.** Wild type (SDA-500) and individuals from isolines A, D, G and I under white light, mCherry and ZsY filters before and 24hrs post blood meal (PBM). Camera settings are detailed in Table S2.

**Table S1**. List of primers used in the study.

| Primer Name | Purpose | Sequence (5’-3’) |
| --- | --- | --- |
| LA7718 | Sequencing | TGCTTGGCAAGCATTGGGTTGGGATGGAAGC |
| LA7719 | PCR | CATCATCGCGATCATCACCTTTGCC |
| LA7720 | PCR | ACGTTGATTGTTTGCCCGCGAGTGG |
| LA7721 | Sequencing | CAGCCCTCCATGTGGTACTTCATGG |
| LA7722 | Sequencing | TAAGCGGCAGGGTCGGAACA |
| LA7723 | Sequencing | GCTCGTATGTTGTGTGGAATTG |
| LA7724 | Sequencing | AAGCTGAAGGTGACCAAG |
| LA7725 | Sequencing | AACCCATGATTATTTGATTAAA |
| LA7726 | Sequencing | CTTTGCGTCAATCAACGAAGC |
| LA7727 | Sequencing | GCACGGGCATGATCTTCT |
| LA7728 | Sequencing | CCCGTGATGAAGAAGATGACCACCA |
| LA7729 | Sequencing | GGCGGATAAAGTTGCAGG |
| LA6798 | Sequencing | gagctggcttGGATAGCGATTCGAGTTAAC |
| LA7703 | PCR | ttttGCTAGCCGTTCGTGATGTTTTCGTGCACTTTC ACGC |
| LA7704 | PCR | ttttGGCGCGCCATCTCGCTTCCGACCAGCATCG CGCATGTG |
| LA6779 | Sequencing | GCTAAGCGAAAGCTAAGCAAA |
| LA6733 | Sequencing | GTGGTTTGTCCAAACTCATC |
| LA6787 | Sequencing | ACGCATGATTATCTTTAACGTACG |

**Table S2.** Visual magnification and exposure (ms) settings for image acquisition.

| **Life Stage**  **(Figure/s)** | **None (brightfield)** | **mCherry** | **ZsYellow** |
| --- | --- | --- | --- |
| **Early Instar Larva (1, S1)** | 32x  99ms | 32x  485ms | 32x  4000ms |
| **Late Instar Larva (1, S1)** | 12.5x  99ms | 12.5x  1000ms | 12.5x  1000ms |

| **Pupa (1)** | 12.5x  99ms | 12.5x  1000ms | 12.5x  1000ms |
| --- | --- | --- | --- |
| **Late Instar Larva (2)** | n/a | 32x  1000ms | 32x  1000ms |
| **Isoline D Adult**  **Male (3)** | 1.5x  99ms | 1.5x  1500ms | 1.5x  1500ms |
| **Isoline A Adult**  **Female (S2)** | 1.5x  99ms | 1.5x  1500ms | 1.5x  1500ms |
| **Isoline D Adult**  **Female (3)** | 1.5x  99ms | 1.5x  1500ms | 1.5x  1500ms |
| **Isoline G Adult**  **Female (S2)** | 1.5x  99ms | 1.5x  6000ms | 1.5x  6000ms |
| **Isoline I Adult**  **Female (S2)** | 1.5x  99ms | 1.5x  1300ms | 1.5x  1300ms |

**Table S3**. Embryonic microinjections

|  | Embryos  injected | G_0_ survivors (%) | G_0_ pools | G_1_ screened  (positives) |
| --- | --- | --- | --- | --- |
| AGG2282 | 399 | 24 (6%) | A (12 G_0_ females)  B (12 G_0_ males) | 373 (101) after which screening was terminated  Not screened |
